# Reassessing the death risk related to probiotics in critically ill patients

**DOI:** 10.1186/s13054-016-1565-1

**Published:** 2016-11-29

**Authors:** Alberto Enrico Maraolo

**Affiliations:** Department of Clinical Medicine and Surgery, Section of Infectious Diseases, University of Naples “Federico II”, Naples, 80131 Italy

Manzanares and colleagues, in their very comprehensive systematic review with meta-analysis [1], conclude that probiotics prove to be a very useful weapon to reduce infections in critically ill patients, although strong recommendation in support of their use cannot be drawn yet. Indeed, they correctly state that publication bias and heterogeneity of the included studies could undermine their conclusions [1]. Another relevant aspect of the paper is the safety profile of probiotics, a well-known matter of debate: no effect was observed upon length-of-stay, diarrhea, and, most of all, mortality [1].

With regard to this crucial point, Fig. 3 in [1] describes the overall effect on hospital mortality, with a risk ratio equal to 0.98 (95% confidence interval 0.82–1.18); obviously the result is not significant but the direction of the effect is in favour of probiotics. It is worth underlining that their Fig. 3 reports a wrong datum about the mortality in the trial by Besselink and colleagues [2], that is 24 out of 152 patients (as correctly reported in Table 2) and not 14 out of 152 in the probiotics arm.

Reassessing the risk ratio with the meta-analytic software ProMeta 3.0, the overall effect becomes 1.02 (95% confidence interval 0.85–1.22), changing the direction of the effect against the use of probiotics, although the result is not significant. The weight of the results stemming from the trial by Besselink and colleagues [2] is, for example, clearly apparent in another systematic review with meta-analysis published in *Critical Care* in 2014 [3].

In spite of their clinical use for a long time, the exact role of probiotics in many therapeutic settings is still not clear, and safety issues in special populations (pregnant women, immunosuppressed, severe underlying diseases) are the main matter of concern [4]. To this purpose, providing the most precise information is fundamental to support clinicians’ decisions.
